# "Discourses to Women on Medical Subjects"
*By Mrs. A. M. Longshore-Potts, M.D, a graduate of the Women's College of Philadelphia, U.S.A. Published by the Author. English Edition.


**Published:** 1888-11-03

**Authors:** 


### "DISCOURSES TO WOMEN ON MEDICAL SUBJECTS."*

BY A CONSULTING PHYSICIAN.

MRS. DR. LONGSHORE-POTTS states that from her earliest experience she has been convinced of the great need for physiological instruction among women, to enable them to live so as to avoid many causes of disease to which they are susceptible. Under this impression she commenced giving lectures throughout the United States, eventually invading the British Isles and the British Colonies. The lectures having been so well received, she has now put the subject-matter into the form of a book, and her object will be gained if the work is the means of leading some to a happier condition of mind and to the possession of better health.

There is undoubtedly much in Dr. Longshore-Potts' book which it would be well if all were acquainted with. On the other hand, there is much in the book which we consider unadvisable should be given forth in a popular form. For example, at the commencement of the work there is an anatomical review, illustrated by plates, of the organs peculiar to females, and the opinion is expressed that the physical structure of both sexes should be understood by the individuals of either. "If a man knew more about the structure of woman it would be to the advantage of women, for then fathers would advise as to the conduct of life of their daughters to the age of maturity" . . . and "brothers would be more thoughtful and considerate for the welfare of their sisters." To this we reply that any attempt to diffuse such knowledge generally must terminate in failure. No person who does not study medicine as a whole can obtain sufficient information of the subjects, not even from Dr. Longshore-Potts' book. The adage, that a little knowledge is a dangerous thing, applies to this perhaps more than to most other matters. As to teaching young females such subjects, we consider more harm than benefit would result. A young female may be metaphored as an electric jar, filled with emotions, and emotions would be wrongly directed if they were caused to dwell on the subjects regarding which Dr. Longshore-Potts would diffuse such general knowledge.

A very good popular work might be made of this book if about half the letterpress and a good many of the illustrations were erased. For instance, we are told, that young girls are like hothouse plants, and are capable of being forced into maturity. Therefore, Dr. Longshore-Potts warns against the use of tight corset, and the too early association with senior companions. Cheerful society, free exercise, good plain nutritious food, loosely-fitting warm garments equally distributed over the body and limbs, plenty of sleep, and judicious choice of books are inculcated, to which the authoress might have added, no forcing of the mental faculties. Now, all this is advice to which exception cannot be taken. But when we find afterwards a chapter on the medical treatment of the various ailments to which young females are more or less liable, we think Dr. Longshore-Potts doth "protest too much." As regards the complaints of older females, Mrs. Longshore-Potts cautions against the use of opium, morphia, or stimulants, for the obtaining of temporary relief from pain; for a habit is thereby likely to be acquired, and drugs or stimulants are taken after the pain which demanded their use has disappeared. Writing of the period known amongst women as the change of life, the authoress rightly observes: "If a woman passes safely over this change, which is fraught with so many peculiar symptoms . . . she may then hope for a period of twenty-five or thirty years of a more undisturbed and peaceful existence than all her previous life has yielded her." It was formerly held that the climateric year of woman is seven times seven, or forty-nine, and there appears to be some truth in this ancient impression.

Every ambitious parent, says Mrs. Longshore-Potts, must desire an intellectual child, as well as one of handsome form and features. The frequent effect on the unborn babe of frightful or unpleasant objects seen by the expectant mother, or even of mental impressions, is a familiar fact; and therefore the authoress states: If the female about to become a mother "desires beauty and symmetry in her offspring, she will have her own mind filled with the ideal." But when Mrs. Longshore-Potts carries on as follows, we really think she does again "protest too much." She writes: "If any particular intellectual stamp is required, the study and contemplation of that one branch should stand paramount in her" (the expectant mother's) "mind. If law is the choice, then she should read and study 'Blackstone,' argue, and talk, and plead for her imaginary client. . . . If the medical profession is the choice for the unborn son or daughter, there should be diligent perusal of some volumes upon the structure of the human system, and study of the functions of the organs. . . . If it is the ministry of the Gospel that is much desired, the mother must devote herself to religion. She should go to church, not to exhibit her new dress or her charming-hat, but in sincerity to worship God. She should pray, but not in public only, that the world might see and hear the prayers she offers, for such services might produce a hypocrite. . . . Music, art, and mechanics may all be thus implanted in the plastic brain-cells of the mysteriously-developing new being."

In this age, when one of the burning questions of the day is "What to do with our boys," it savours rather too much of counting chickens before they are hatched to fix the profession of a babe yet unborn, and before its sex even is an established fact! No doubt intellectual features are hereditary to some extent, and, like physical features, they crop out in future generations. But even in the laws of heredity, equally with most other laws, there is a glorious uncertainty, and it will be quite sufficient to direct the mind of a child to his future employment when the proper period for special education arrives. The following is a delicious passage: "The pregnant mother, with several children to feed and clothe on limited means, must necessarily think how it is to be accomplished; her son must go to school, but how can she furnish him with neat, sound garments to fit him to associate with others perhaps more favoured with worldly comforts? After much reflection, she looks over cast-off garments which her husband long since ceased to wear. Among them are his wedding trousers, soiled, torn, and much the worse for long and perpetual use. Now in this emergency a thought flashes into her mind, and makes her very happy—out of these trousers she will contrive a suit for her little son. With pleasure she carefully washes, rips, and presses all the parts, and with her paper patterns tries to make the pieces all come out. First the paper is laid this way, then that; with a corner piece put on here, and a rent darned there, she thinks and plans how to get that tiny suit. At length she triumphs, and with delight she fits the garments to her son. Now the boy can go to school; and while she was planning, constructing, and speculating over that dusty, musty cloth, she was weaving real genius and mechanism into the developing mind of her coming child; and if education and circumstances favour such a result, that child when matured will be a master mechanic of rare ability." That is just it: "if education and circumstances favour such a result." And herein Mrs. Longshore-Potts somewhat contradicts her previous assertion, that the woman by reading law, physic, or divinity, will produce a lawyer, a physician, or a divine.

As observed above, the book might be made a good one if much were omitted. As it now stands, we cannot advise females to read it.

* By Mrs. A. M. Longshore-Potts, M.D., a graduate of the Women's College of Philadelphia, U.S.A. Published by the Author. English Edition.

